# Characterization of *Weissella viridescens* UCO-SMC3 as a Potential Probiotic for the Skin: Its Beneficial Role in the Pathogenesis of Acne Vulgaris

**DOI:** 10.3390/microorganisms9071486

**Published:** 2021-07-13

**Authors:** Marcela Espinoza-Monje, Jorge Campos, Eduardo Alvarez Villamil, Alonso Jerez, Stefania Dentice Maidana, Mariano Elean, Susana Salva, Haruki Kitazawa, Julio Villena, Apolinaria García-Cancino

**Affiliations:** 1 Laboratory of Bacterial Pathogenicity, Faculty of Biological Sciences, University of Concepcion, 4030000 Concepcion, Chile; marespinozamonje@gmail.com (M.E.-M.); jorgcampos92@gmail.com (J.C.); ajerez@udec.cl (A.J.); 2 Laboratory of Immunobiotechnology, Reference Centre for Lactobacilli (CERELA-CONICET), CP 4000 Tucuman, Argentina; ealvarez@cerela.org.ar (E.A.V.); stefi.dentice@gmail.com (S.D.M.); melean@cerela.org.ar (M.E.); ssalva@cerela.org.ar (S.S.); 3 Food and Feed Immunology Group, Laboratory of Animal Food Function, Graduate School of Agricultural Science, Tohoku University, Sendai 980-8572, Japan; 4 Livestock Immunology Unit, International Education and Research Center for Food Agricultural Immunology (CFAI), Graduate School of Agricultural Science, Tohoku University, Sendai 980-8572, Japan

**Keywords:** *Weissella viridescens* UCO-SMC3, probiotic, immunobiotic, skin immunity, acne vulgaris, *Cutibacterium acnes*, *Staphylococcus aureus*, skin inflammation

## Abstract

Previously, we isolated lactic acid bacteria from the slime of the garden snail *Helix aspersa* Müller and selected *Weissella viridescens* UCO-SMC3 because of its ability to inhibit in vitro the growth of the skin-associated pathogen *Cutibacterium acnes*. The present study aimed to characterize the antimicrobial and immunomodulatory properties of *W. viridescens* UCO-SMC3 and to demonstrate its beneficial effect in the treatment of acne vulgaris. Our in vitro studies showed that the UCO-SMC3 strain resists adverse gastrointestinal conditions, inhibits the growth of clinical isolates of *C. acnes*, and reduces the adhesion of the pathogen to keratinocytes. Furthermore, in vivo studies in a mice model of *C. acnes* infection demonstrated that *W. viridescens* UCO-SMC3 beneficially modulates the immune response against the skin pathogen. Both the oral and topical administration of the UCO-SCM3 strain was capable of reducing the replication of *C. acnes* in skin lesions and beneficially modulating the inflammatory response. Of note, orally administered *W. viridescens* UCO-SMC3 induced more remarkable changes in the immune response to *C. acnes* than the topical treatment. However, the topical administration of *W. viridescens* UCO-SMC3 was more efficient than the oral treatment to reduce pathogen bacterial loads in the skin, and effects probably related to its ability to inhibit and antagonize the adhesion of *C. acnes*. Furthermore, a pilot study in acne volunteers demonstrated the capacity of a facial cream containing the UCO-SMC3 strain to reduce acne lesions. The results presented here encourage further mechanistic and clinical investigations to characterize *W. viridescens* UCO-SMC3 as a probiotic for acne vulgaris treatment.

## 1. Introduction

The skin is the largest organ in the human body and functions as the first line of defense by providing a protective barrier between the external environment and the internal tissues of the host. Recent studies have shown that the skin’s microbiota plays a fundamental role in protecting the host by producing antimicrobial and immunomodulatory compounds that help to prevent the invasion of pathogens and the excessive growth of opportunistic microorganisms [1,2,3]. Alterations of the natural barrier given by the skin can lead to the appearance of local pathologies or diseases that can affect deeper organs. Among the most common skin diseases is acne vulgaris, which affects 90% of the adolescent population worldwide and can persist into adulthood in 12–14% of patients [4]. Thus, this pathology can affect patients from their school-age to adulthood [5].

*Cutibacterium acnes* (formerly known as *Propionibacterium acnes*) play a key role in the pathogenesis of acne [6]. This Gram-positive bacterium is part of the normal microbiota of the skin, and although it is present at a low level on the epidermal surface, it constitutes the dominant bacteria in the sebaceous follicles [7]. In patients with acne, *C. acnes* is found in abnormally high numbers within sebaceous follicles [6,7]. Moreover, the observation that the reduction of *C. acnes* loads in the skin correlates with clinical improvements that further emphasize the role of this bacterium in acne vulgaris [8]. Of note, the pathophysiology of acne has undergone a paradigm shift in recent years, changing our understanding of the role of *C. acnes* in this disease. Now it is considered that a combination of a dysbiosis of the skin microbiome and the alteration in the balance of the different *C. acnes* phylotypes are involved in acne development rather than the hyperproliferation of this bacterium [7]. In fact, metagenomics studies have reported a similar relative abundance of *C. acnes* in healthy individuals and in patients with acne [6]. However, the proportion of different *C. acnes* phylotypes were different when acne patients were compared with healthy persons. A predominance of *C. acnes* phylotype IA1 [9] and strains carrying extra virulence genes [10] have been found in acne samples. On the other hand, acne is a chronic inflammatory skin condition, and, therefore, the immune system is an important player in the development of the disease. During its multiplication, *C. acnes* produce hydrolytic enzymes including hyaluronidases, proteases, and lipases that damage skin tissues, leading to the stimulation of cutaneous inflammation [11]. It was shown that *C. acnes* strains isolated from acne samples produce significantly higher levels of porphyrins, which are metabolites with the ability to stimulate the generation of reactive oxygen species and the production of inflammatory factors by keratinocytes [12]. The local inflammation is amplified leading to the stimulation of local immune cells and the further release of chemotactic and inflammatory mediators that attract immune cells to the infected sebaceous follicles causing chronic inflammation.

The treatment of acne vulgaris includes the use of long-term therapies, particularly with antibiotics including erythromycin, clindamycin, and tetracycline, which have generally good results. However, due to the side effects produced by the therapeutic drugs, patients generally do not adhere completely to the treatment and end up abandoning it. In addition, oral antibiotics for acne treatment have been recently associated with the development of antimicrobial resistance [13,14]. For these reasons, different alternatives for acne treatment are being searched including the use of beneficial microorganisms. In this regard, topical bacteriotherapy was first proposed as a treatment for skin diseases in 1912, when a topical application of *Lactobacillus bulgaricus* was reported to have beneficial effects on acne and seborrhea [15]. Following the rise of probiotic research, numerous topical microbial formulations have been proposed in recent years to correct skin dysbiosis [16]. Topical probiotics have shown remarkable efficacy in clinical trials aimed to evaluate their effect on acne, atopic dermatitis, and rosacea [17]. Therefore, in the last decade, commercially available topical probiotics for application to the skin have gained great popularity. In addition, orally administered probiotics interventions in dermatologic diseases including acne have similarly gained significant attention [1]. However, it should be noted that no in-depth studies have been conducted to characterize the cellular and molecular mechanisms associated with the beneficial effects of these probiotics. These studies are necessary to provide solid scientific bases that allow proposing probiotics as a real alternative for the prevention or treatment of skin pathologies.

The slime of the common garden snail, *Helix aspersa* Müller, has been reported to have various cosmetic and beneficial properties for skin health [18,19]. However, little is known about the microbial populations of the snail’s slime or gastrointestinal tract. A study showed that the gastrointestinal microbiota of healthy snails has a predominance of *Pediococcus*, *Lactobacillus,* and *Lactococcus* genera [20]. Interestingly, this study demonstrated that snails suffering from gastrointestinal disturbances had an elevated proportion of *Klebsiella*, *Citrobacter,* and *Enterobacter* species together with a reduced presence of *Lactobacillus* and *Lactococcus* strains. These results indicated the presence of lactic acid bacteria (LAB) as normal members of the snail microbiota, which could confer beneficial effects to the host.

We hypothesized that the snail microbiota can be involved in the beneficial effects of slime on the human skin, and therefore, we have recently isolated and investigated LAB from the slime of the garden snail *H. aspersa* Müller [21]. Of note, among the strains evaluated, *Weissella viridescens* UCO-SMC3 showed the ability to inhibit in vitro the growth of *C. acnes*. In addition, the genomic characterization of the UCO-SMC3 strains revealed the presence of several genes associated with the capacity of efficiently colonizing the skin tissue [21]. These results encourage more in-depth studies of this strain in order to characterize it as a potential probiotic for application to the skin. Then, the present study aimed to carry out a detailed characterization of the antimicrobial and immunomodulatory properties of *W. viridescens* UCO-SMC3 and to demonstrate its beneficial effect in the treatment of acne vulgaris.

## 2. Materials and Methods

### 2.1. Isolation and Identification of Weissella viridescens UCO-SMC3

The isolation of *W. viridescens* UCO-SMC3 was carried out from adult snails corresponding to the *Helix aspersa* Müller species as previously described [21]. Briefly, the snails were fasted for 12 h and then stimulated to obtain mucous secretion in a biosecurity cabinet. This secretion was collected and seeded on Man-Rogosa-Sharpe (MRS, Oxoid, Cambridge, United Kingdom) agar for the isolation of LAB at 37 °C and 10% CO_2_ for 48 h. For its identification, a single colony of the UCO-SMC3 strain was selected, which was cultivated for 12 h at 37 °C in MRS broth. The genomic DNA of *W. viridescens* UCO-SMC3 was obtained following the procedure described in [22] and the entire genome was sequenced with the paired-end reading length sequencing protocol of 2 × 150 bp of the Illumina MiSeq platform [21]. *W. viridescens* UCO-SMC3 genome accession number is RHGY00000000.

### 2.2. Glass Adherence

*W. viridescens* UCO-SMC3 and the control strain *Limosilactobacillus fermentum* (Basonym: *L. fermentum*) UCO-979C were grown in MRS broth medium as described above. Bacterial cells were harvested by centrifugation and suspended in 20 mL of MRS broth at a cell density of 2 on the McFarland scale and incubated for 1 h. Subsequently, a sterile slide was introduced to each culture and incubated for 4 h. Each slide was then stained with 0.1% of crystal violet for 5 min, excess staining was removed with 70% alcohol for 30 s and dried at room temperature. The slides were visualized in an optical microscope (100×) and the strains were classified as non-adherent (less than 20 bacteria per field), moderately adherent (20–50 bacteria per field), and strongly adherent (over 50 cells per field) [23].

### 2.3. Culture of HaCat Cells and Adherence Assay

The adherence of *W. viridescens* UCO-SMC3 was evaluated by an in vitro assay using the HaCat cell line (CLS Cell Lines Service, 300493, Eppelheim, Germany), which are epidermal keratinocytes. For this purpose, the HaCat cells were cultured in a 24-well plate that contained sterile round coverslips (Deckgläser coverslips, Eppelheim, Germany, 16 mm). The cells were cultured with DMEM supplemented with 10% fetal bovine serum (Biological Industries, Cromwell, CT, USA) and 1% antimicrobial solution (Biological Industries) at 37 °C, 5% CO_2_ until a monolayer was formed (approximately 4 days). Then, the cells (2.5 × 10^6^ per well) were washed twice with PBS and were treated with 500 μL of the bacterial suspension at a concentration of 0.5 Mc Farland (1.5 × 10^8^ CFU/mL, MOI = 30) for 5 h. The cells were washed twice with PBS, fixed with 700 μL of 2.5% glutaraldehyde, and stored at 4 °C for analysis by scanning electron microscopy. The samples were visualized using an Auto-scan model U1 scanning electron microscope (ETEC Corporation, Chile) at the Center for Microscopy and Spectroscopy, University of Concepción, Chile.

On the other hand, cells obtained in the same way were fixed with 500 μL of methanol for 5 min and stained with 200 μL of crystal violet per 1 min for their visualization by light microscopy. Each sample was analyzed under an Olympus^®^ IX81 light microscope using a 100× immersion objective to detect bacteria adhering to the cells. The level of adherence was measured considering the following criteria: not adhered (≤5 bacteria/100 cells); adherent (5–40 bacteria/100 cells), and strongly adhered (>40 bacteria/100 cells).

### 2.4. Resistance to Gastric Conditions

Acidic tolerance was studied by transferring 100 μL of a 24-h liquid culture of the *W. viridescens* UCO-SMC3 strain or the control, *L. fermentum* UCO-979C strain, to 10 mL of MRS broth adjusted to pH 2 or 3 with HCl. The cultures were incubated at 37 °C and 10% CO_2_ for 24 h. Aliquots were removed at 0, 1, 2, 3, and 24 h of incubation to determine the number of cultivable bacteria by the micro-drop method. Bile tolerance was tested similarly. For this purpose, 10 mL of MRS broth were supplemented with bile salts (Oxgall, Lansing, MI, USA) at 1.5% and 2% without adding HCl [24].

### 2.5. Antibiotic Susceptibility

The antibiotic susceptibility of *W. viridescens* UCO-SMC3 was evaluated using the antibiotics (Oxoid™, Thermo Fisher Scientific, Waltham, MA, USA): streptomycin, etrithromycin, amikacin, gentamicin, ampicillin, cefuroxime, penicillin G, sulfatrimethoprim, cefotaxin, amoxicillin, levofloxacin, chloramphenicol, clarithromycin, neomycin, ciprofloxacin, rifampicin, vancomycin, and tetracycline. Susceptibility was determined by an agar diffusion test [25]. The criterion of Georgieva et al. [26] was used for the classification of strains as susceptible or resistant to each antibiotic.

### 2.6. Hemolysis and Gelatinase Activities Detection

*W. viridescens* UCO-SMC3 was grown on Columbia agar supplemented with 5% human or horse blood at 37 °C and 10% CO_2_ for 48 h. The presence or absence of hemolysis was analyzed, and in the first case, α-hemolysis and β-hemolysis were discriminated [27]. For the evaluation of gelatinase activity, the UCO-SMC3 strain was cultivated in nutritive gelatin for microbiological use at 37 °C and 10% CO_2_ for 48 h. After bacterial growth, the tubes were refrigerated and their gelation was analyzed [28].

### 2.7. Cytotoxicity Assay on HaCat Cells

The potential cytotoxicity of *W. viridescens* UCO-SMC3 on the HaCat cell line was evaluated by the colorimetric method using the LDH-CytoxTM Assay kit according to the manufacturer’s instructions. Preliminary tests allowed optimizing the cell concentration to 12,000 cells/mL. Briefly, 100 μL of cell suspension was added to each well of a 96-well plate at a concentration of 12,000 cells per well. Cells were incubated overnight at 37 °C and 5% CO_2_. Three bacterial concentrations were tested: 1 × 10^9^, 1 × 10^8^ and 1 × 10^7^ CFU/mL. HaCat cells stimulated with the bacteria were incubated at 37 °C for 24 h. Finally, the percentage cytotoxicity was evaluated and calculated according to the formula established by the manufacturer.

### 2.8. Hydrogen Peroxide (H_2_O_2_) Production

For the determination of H_2_O_2_ production, the semi-quantitative method described by Felten et al. [29] was performed. Briefly, *W. viridescens* UCO-SMC3 was inoculated on MRS agar supplemented with 3, 3′, 5, 5′ tetramethylbenzidine and peroxidase, and incubated for 48 h at 37 °C, 10% CO_2_ to record changes in the color of the medium. *Lacticaseibacillus rhamnosus* GG was used as a positive control. For the interpretation of the results, the following criteria were applied: white colonies (negative production, −), light blue colonies (low production, +), blue colonies (moderate production, ++), and dark blue colonies (strong production, +++) [29].

### 2.9. Lactic Acid and Bacteriocin Detection

The determination of lactic acid production was performed by spectrophotometry by the enzymatic method using the L-Lactic Acid (L-Lactate) Assay (Megazyme, Butzbach, Germany) kit. *W. viridescens* UCO-SMC3 was grown in MRS medium at pH 8.0 and 10% CO_2_ for 48 h. The samples were then filtered using 0.22 μM filters and analyzed according to the manufacturer’s conditions.

Bacteriocin detection was carried out using a conventional PCR for six bacteriocin genes present in different LAB species, using the primers described in previous works: pediocin [30], plantarazin A [30], sakasin P [31], acidocin [32], and salivaricin B [33]. For the identification of bacteriocin ABP118, different primers were used in relation to α and β subunit of bacteriocin [34] generating amplifications of 277 bp and 340 bp, respectively. The PCR conditions were carried out according to those described by the cited works, with minor modifications. The reaction was performed in a volume of 25 μL, which consisted in: 12.5 μL of PrimerMix Takara, 1 μL of the forward primer, 1 μL of the reverse primer, 8.5 μL of PCR grade water, and 2 μL of bacterial DNA. The amplification products were analyzed by electrophoresis in agarose gels at 1 and 2% *w/v*, and were visualized in a UV transilluminator (ENDUROTM GDS). The BAGEL 4 platform and blastp were used to search for bacteriocins in the UCO-SCM3 genome.

### 2.10. Microbicidal Activity on C. acnes and S. aureus

*C. acnes* ATCC 6919 and *C. acnes* 6P2 (phylotype IA1) were grown in Reinforced Clostridial Medium (RCM) and *S. aureus* ATCC 6538 in Trypticase Soy Agar (TSA). Inhibitory activity was performed using the radial diffusion methodology described by Sgouras et al. [25]. Wells of 6 mm diameter were made with a sterile punch in plates with 20 mL of Müeller Hinton agar (Difco™, Buenos Aires, Argentina). Subsequently, the plates were inoculated with a suspension of *C. acnes* or *S. aureus* at a McFarland concentration No. 0.5 (1.5 × 10^8^ CFU/mL). In each well, 50 μL of the *W. viridescens* UCO-SMC3 inoculum was deposited at a cell density of McFarland No. 2. The plates were incubated at 37 °C for 24 h for the *S. aureus* assay and 72 h for the test with *C. acnes*, both at 10% CO_2_. The bactericidal activity of the probiotic strain *L. fermentum* UCO-979c and the strain *W. viridescens* CH (isolated from the faces of garden snails) against *C. acnes* and *S. aureus* were performed in parallel for comparisons. To evaluate the inhibition, the criterion of Gaudana et al. [35] was used, which discriminates: no inhibition (diameter < 1 mm), mild inhibition (diameter 1–2 mm), strong inhibition (diameter 2–5 mm), and remarkable strong inhibition (diameter > 5 mm). Final values of inhibition diameters were obtained by subtracting the well size (6 mm) from the measured zone of inhibition. All experiments were carried out in triplicate.

### 2.11. Antagonistic Activity on the Adhesion of C. acnes and S. aureus in HaCat Cells

HaCat cells (2000/mL) were seeded in 24-well plates and cultured at 37 °C and 5% CO_2_ until reaching confluence. Then, three separate tests were performed to evaluate the exclusion, competition, and displacement capacity of *W. viridescens* UCO-SMC3 on the adhesion of pathogens *C. acnes* and *S. aureus*.

For these tests, 1.5 × 10^8^ CFU/mL of the UCO-SMC3 strain and 1.5 × 10^7^ CFU/mL of the pathogenic strains *C. acnes* or *S. aureus* were used. The cells were exposed to *W. viridescens* UCO-SMC3 1 h before the addition of *C. acnes* or *S. aureus* to evaluate the exclusion capacity. On the other hand, to evaluate the ability of competition, *W. viridescens* UCO-SMC3 was added to the cells at the same time as the pathogenic bacteria, while the UCO-SMC3 strain was added 30 min after the addition of *C. acnes* or *S. aureus* to assess displacement. After the incubation of cells with the bacterial strains, the wells were trypsinized to subsequently evaluate the bacterial growth. *W. viridescens* UCO-SMC3 counts were performed in culture plates of MRS agar using the microdroplet technique. In parallel, Reinforced Clostridial and Trypticase medium were used to obtain the counts of *C. acnes* and *S. aureus*, respectively.

### 2.12. Protection Against C. acnes Infection In Vivo

Female 6-week-old Balb/c mice were obtained from the closed colony kept at CERELA (Tucuman, Argentina). They were housed in plastic cages with a controlled room temperature (22 ± 2 °C temperature, 55 ± 2% humidity) and were fed *ad libitum* a conventional balanced diet. Animal welfare was ensured by researchers and specially trained staff in animal care and handling at CERELA. Animal health and behavior were monitored twice a day. This study was carried out in strict accordance with the recommendations in the Guide for the Care and Use of Laboratory Animals of the Guidelines for Animal Experimentation of CERELA. The CERELA Institutional Animal Care and Use Committee prospectively approved this research under the protocol BIOT-CRL-19.

On day 1, the dorsal hair of all mice was shaved with an electric clipper to obtain a square of 1.5 × 2.0 cm in the back of each animal. Then, mice were randomly divided into three groups (Supplementary Figure S1): with no UCO-SMC3 treatment (control group); treated topically with the UCO-SMC3 strain (cutaneous UCO-SMC3 group) or treated orally with the UCO-SMC3 strain (oral UCO-SMC3 group). The cutaneous UCO-SMC3 group received 100 μL of a suspension containing *W. viridiscens* UCO-SMC3 (1 × 10^8^ bacteria per mL of sterile PBS) on the back while the oral UCO-SMC3 group received 10^8^ *W. viridiscens* UCO-SMC3 in 200 μL of sterile non-fat milk (10%) by gavage. In the UCO-SMC3 groups, the bacterium was administered daily for 10 days. On day 11, these mice and the untreated control group were challenged with *C. acnes* 6P2. The pathogen was administered through a subdermal injection of 100 μL of a solution containing 1 × 10^8^ CFU. All the infected animals were sacrificed on day 16 (5 days after *C. acnes* infection).

For *C. acnes* counts in skin samples, a previously described protocol was used [36,37]. Briefly, skin samples (1.5 × 2.0 cm) from the infection site were aseptically removed and placed in homogenization vials with 2.0 mL of PBS solution, weighed, and homogenized. The homogenates were serially diluted and plated anaerobically on RCM agar plates for the enumeration of *C. acnes*. Tissue weights were used to calculate the log CFU per gram of skin tissue.

### 2.13. Determination of Blood Leukocytes Counts and Serum Cytokines

The total number of leukocytes was determined with a hemocytometer. Differential cell counts were performed by counting 200 cells in blood smears stained with May Grunwald-Giemsa. The concentration of cytokines was determined in blood samples of UCO-SMC3-treated and control mice. Blood samples were obtained through cardiac puncture at the end of each treatment and collected in heparinized tubes. Tumor necrosis factor α (TNF-α), interferon-γ (IFN-γ), and interleukin 1β (IL-1β), IL-4, IL-10, and IL-17 concentrations in serum were measured with commercially available enzyme-linked immunosorbent assay (ELISA) kits following the manufacturer’s recommendations (R&D Systems, Minneapolis, MN, USA).

### 2.14. Flow Cytometry Studies in Lymphoid Nodes

Facial lymph nodes (FLN) and axillary lymph nodes (ALN) were collected and mechanically disaggregated. A single-cell suspension from the FLN and the ALN of each mouse was obtained by gently passing the collected tissue through a tissue strainer with PBS with 2% FCS (FACS buffer). Cell suspensions were subjected to red blood cells lysis (Tris-ammonium chloride, BD PharMingen, Frankiln Lakes, NJ, USA) flowed by counting on a hemacytometer. The viability of cells was assessed by trypan blue exclusion. Cell suspensions were pre-incubated with anti-mouse CD32/CD16 monoclonal antibody (Fc block) for 30 min at 4 °C. Cells were incubated with the antibody mixes for 30 min at 4 °C and washed with FACS buffer. The following antibodies from BD Biosciences were used: FITC-labeled anti-mouse MHC-II, FITC-labeled anti-mouse CD3, biotinylated anti-mouse CD4, FITC-labeled anti-mouse CD25, and PE-labeled anti-mouse CD11c antibodies. Streptavidin-PerCP was used as a second-step reagent. Flow cytometry was performed using a BD FACSCalibur^TM^ flow cytometer (BD Biosciences, Frankilin Lakes, NJ, USA) and data were analyzed using FlowJo software (TreeStar, BD Biosciences, NJ, USA).

### 2.15. Development of Novobase II Cream with W. viridescens UCO-SMC3

The formulation of the cream containing the UCO-SMC3 strain was prepared with the advice of the Department of Pharmacy of the Faculty of Pharmacy, (University of Concepción, Chile). The novobase II formulation was prepared with 5% stearyl alcohol, 10% cetyl alcohol, 1% sodium lauryl sulfate, 10% propylene glycol, and distilled water. No preservatives were used to prevent the death of *W. viridescens* UCO-SMC3. The final concentration of bacteria was 1 × 10^8^ UFC/mL of cream.

The bacterial strain was incorporated into the cream in lyophilized form. For this, *W. viridescens* UCO-SMC3 was cultivated in 1 L of MRS medium at 37 °C and 10% CO_2_ for 24 h. For lyophilization, the service of Laboratorios Pasteur S.A. (Concepción, Chile) was hired.

### 2.16. Clinical Pilot Trial

For the pilot trial aimed at evaluating the ability of the cream containing *W. viridescens* UCO-SMC3 to improve acne, women were recruited for their high adherence to dermo-cosmetic treatments. The participants were students recruited from the University of Concepcion who presented with acne. The selection was made by examination of the lesions. Inclusion criteria for the trial were determined as follows: age 18 to 30 years, not having any type of acne treatment, and not having other skin disorders. Thus, 13 volunteers with acne were selected. In addition, 5 acne-free volunteers were incorporated into the study.

Each volunteer signed an informed consent to enter the study and was given an explanatory sheet with the conditions for using the cream containing the UCO-SMC3 strain. The instructions for use of the cream indicated: wash the face with a neutral soap provided in the study, apply a small amount of cream at night for a period of 5 weeks, and store the cream in a refrigerator (4 °C) due to the absence of preservatives. Each volunteer was evaluated once a week for the photographic record of the evolution of the lesions. A new cream was provided at each appointment, to avoid contamination of the prototype.

Considering that acne lesions may vary in number and extension during the natural course of the disease, various measurements have been developed to assess the clinical severity, with the clinical examination and photographic documentation the most commonly used [38,39,40]. For the quantification of the clinical examination, a score was determined considering a grading system to estimate the extent of involvement and lesion counting. The improvement of the lesions from the beginning to the end of the study was quantified considering a high (4 points), moderate (3 points), mild (2 points), or no (1 point) reduction of inflammation, and estimating the extent of involvement. Similarly, the variations in lesion counts were scored as high (4 points), moderate (3 points), mild (2 points), or no (1 point) reduction. A final score was calculated for each patient with a maximum of 8 points (high reduction of inflammation and lesions counts) and a minimum of 2 (no reduction of inflammation and lesions counts).

### 2.17. Statistical Analysis

Experiments were performed in triplicate and results were expressed as the mean ±SD. For the comparison of two groups, the Student’s *t*-test was used. For the comparison of more than two groups, a one-way analysis of variance (ANOVA) was performed followed by the Tukey’s test. In all cases, a level of significance of *p* < 0.05 was considered.

## 3. Results

### 3.1. Adherent Capacity of W. viridescens UCO-SMC3

**Figure 1:**
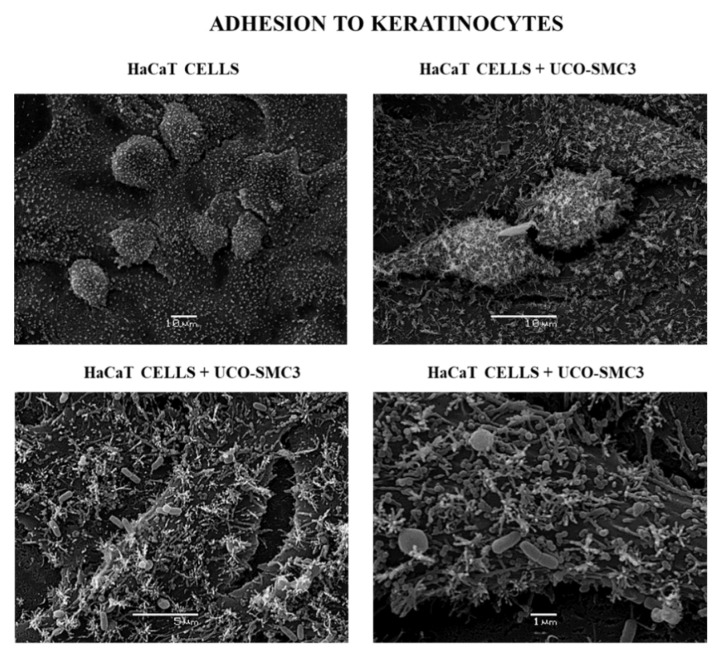
Adherence of *Weissella viridescens* UCO-SMC3 on the HaCat cell line (keratinocytes). Electron microscopy photographs show *W. viridescens* UCO-SMC3 adhered to the surface of HaCat epidermal keratinocytes in different amplifications.

The adhesive ability of the UCO-SMC3 strain was first evaluated, considering that this characteristic would be an important requirement for the bacteria to exert its beneficial properties on the skin. Then, an abiotic surface represented by the glass and epithelial cells of the skin was considered for the experiments. It was observed that the UCO-SMC3 strain is moderately adherent on an abiotic surface, with a count greater than 20 bacterial cells per visual field. Regarding its adherence to HaCat epidermal keratinocytes, it was possible to detect that *W. viridescens* UCO-SMC3 turned out to be strongly adherent, with a count greater than 40 cells per visual field (Supplementary Figure S2). This ability to efficiently adhere to skin epithelial cells was confirmed by electron scanning microscopy (Figure 1).

### 3.2. Resistance of W. viridescens UCO-SMC3 to Gastrointestinal Conditions

**Figure 2:**
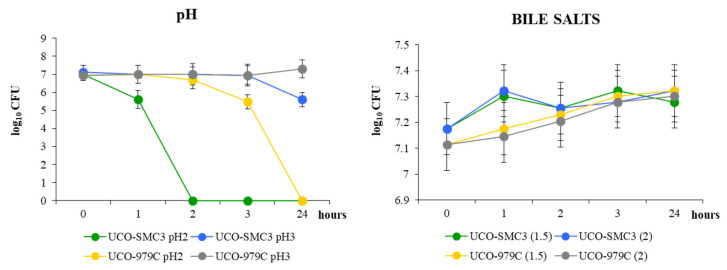
Growth curves of *Weissella viridescens* UCO-SMC3 and the probiotic strain *Limosilactobacillus fermentum* UCO-979C in MRS broth with pH adjusted to 2 and 3 by adding hydrochloric acid or supplemented bile salts at concentrations of 1.5 and 2%.

Taking into account that some studies have reported the ability of probiotic microorganisms administered orally to exert beneficial effects on the skin [40,41,42], we decided to study the ability of *W. viridescens* UCO-SMC3 to resist simulated gastrointestinal conditions *in vitro*. These studies were carried out in comparison with the probiotic strain *L. fermentum* UCO-979C [43,44]. *W. viridescens* UCO-SMC3 was capable of growing at pH 3; however, its growth was significantly reduced in the medium of pH 2 (Figure 2). This behavior was similar to that observed for the UCO-979C strain although lactobacilli survived for a more prolonged time at pH 2. None of the strains could be recovered at 24 h in the MRS medium at pH 2.

The study of bile salt tolerance showed that *W. viridescens* UCO-SMC3 was able to resist both concentrations of bile salts tested: 1.5% and 2% (Figure 2). The presence of bile salts did not significantly modify the viable bacteria counts for the UCO-SMC3 strain. The ability to tolerate bile salts of *W. viridescens* UCO-SMC3 was similar to the observed in control strain UCO-979C.

### 3.3. Innocuousness of W. viridescens UCO-SMC3

To evaluate the safety of *W. viridescens* UCO-SMC3, its antibiotic resistance, hemolytic capacity, and gelatinase activity were studied. The UCO-SMC3 strain was susceptible to most of the antibiotics tested, showing resistance to only two of them: sulfatrimethoprim and ciprofloxacin (Table 1).

The hemolysis test determined that *W. viridescens* UCO-SMC3 is γ-hemolytic, according to the criteria of Peres et al. [27]. The studies also revealed that the UCO-SMC3 strain also does not exhibit gelatinase activity. In addition, the toxicity test of the UCO-SMC3 strain showed that only 1.3% of the HaCat cells lost their viability after a period of 24 h and with a bacterial concentration of 10^9^ CFU/mL. These results indicate that *W. viridescens* UCO-SMC3 does not show significant cytotoxicity on the human keratinocyte cell line.

### 3.4. Antimicrobial Activity of W. viridescens UCO-SMC3

**Figure 3:**
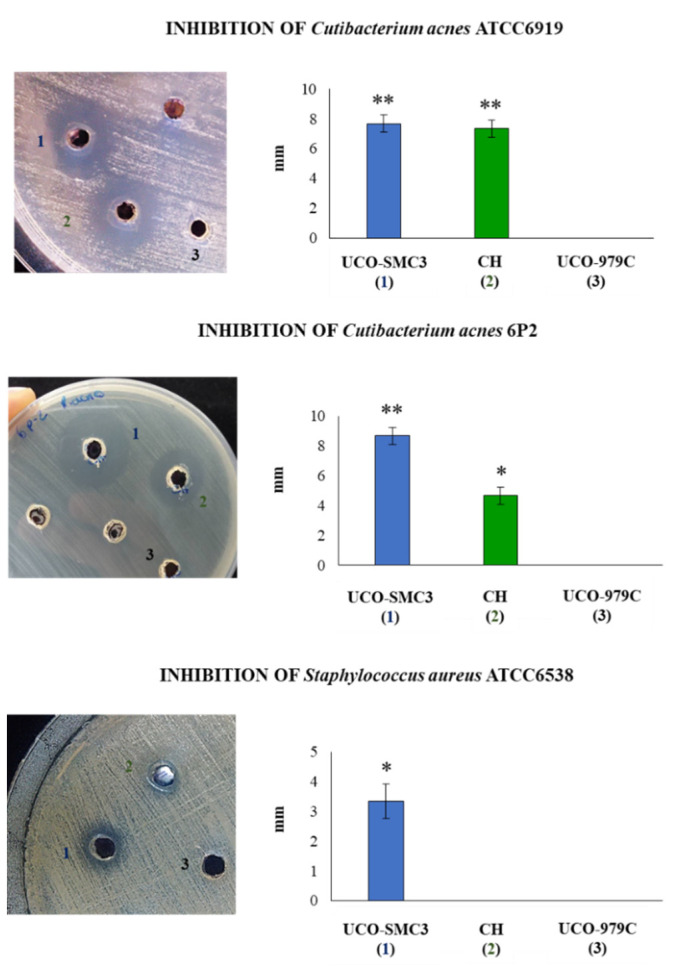
Bactericidal activity of *Weissella viridescens* UCO-SMC3 (**1, blue**), *W. viridescens* CH (**2, green**), and *Limosillactobacillus fermentum* UCO-979C (**3, black**) on skin-associated pathogens. Inhibition capacities of the strains were tested with *Cutibacterium acnes* ATCC 6919, the clinical isolate *C. acnes* 6P2 (phylotype IA1), and *Staphylococcus aureus* ATCC 6538. (*) *p* < 0.05, (**) *p* < 0.01.

*W. viridescens* UCO-SMC3 showed a high microbicidal activity when confronted with *C. acnes* ATCC6919 and with the clinical strain *C. acnes* 6P2 phylotype IA1 (Figure 3). A strong inhibition against *S. aureus* ATCC 6538P (Figure 3) could also be observed according to the criteria of Gaudana et al. [35]. Interestingly, the probiotic strain *L. fermentum* UCO-979C inhibit *S. aureus* but it did not show an inhibitory effect on the *C. acnes* strains (Figure 3).

In addition, we evaluated whether another *W. viridescens* strain isolated from the faces of garden snails could inhibit the growth of *C. acnes* and *S. aureus*. As shown in Figure 3, *W. viridescens* CH was capable of inhibiting the growth *C. acnes* ATCC6919 and *C. acnes* 6P2. However, its ability to reduce the growth of the clinical isolate 6P2 was significantly lower than that observed for *W. viridescens* UCO-SMC3. Furthermore, *W. viridescens* CH was not capable of affecting the growth of *S. aureus* (Figure 3). These results indicate a specific-dependent capacity of snail *Weissella* strains to inhibit acne-associated pathogens.

We also evaluated several microbial molecules that could be involved in the antimicrobial activities of *W. viridescens* UCO-SMC3. The strain was able to produce lactic acid in a concentration of 1.55 ± 0.01 g/L, a level that was comparable to that observed in the probiotic strain *L. fermentum* UCO-979C (1.39 ± 0.09). The semi-quantitative evaluation of H_2_O_2_ production revealed that *W. viridescens* UCO-SMC3 is a very good producer of this molecule (+++) surpassing the probiotic bacteria *L. fermentum* UCO-979C (++) and *L. rhamnosus* GG (+) in this capacity (Supplementary Figure S3). The bacteriocins pediocin, plantarazin A, sakasin P, acidocin, salivaricin B, and bacteriocin ABP118 were searched by using the specific primers. None of these bacteriocins were found in *W. viridescens* UCO-SMC3 (Supplementary Figure S4). These results were corroborated by analyzing the complete genome of the UCO-SCM3 strain.

### 3.5. Antagonistic Activity of W. viridescens UCO-SMC3 against Skin Pathogens

**Figure 4:**
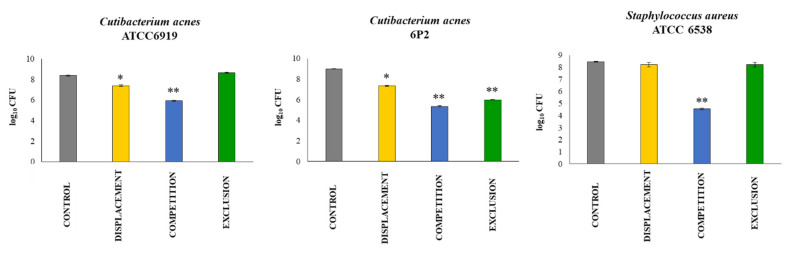
Antagonistic activity of *Weissella viridescens* UCO-SMC3 against skin-associated pathogens. Displacement, competition, and exclusion capacities of the UCO-SMC3 strain were tested with *Cutibacterium acnes* ATCC 6919, the clinical isolate *C. acnes* 6P2 (phylotype IA1), and *Staphylococcus aureus* ATCC 6538. Statistically different compared to control group (*) *p* < 0.05, (**) *p* < 0.01.

*W. viridescens* UCO-SMC3 showed an antagonistic effect against strains of *C. acnes* in HaCat cells (Figure 4). The UCO-SMC3 strain was able to displace and compete with the clinical strain *C. acnes* 6P2 as with the control strain ATCC6919. Furthermore, it was observed that *W. viridescens* UCO-SMC3 was able to exert an exclusion effect on *C. acnes* 6P2. On the other hand, it was also observed that the UCO-SMC3 strain is capable of exerting an antagonistic effect against *S. aureus* ATCC6538 by competition (Figure 4). In fact, the UCO-SMC3 strain was able to reduce the pathogenic strain by 3 logs, which according to Pearson’s criterion [45] corresponds to bacterial death.

**Figure 5:**
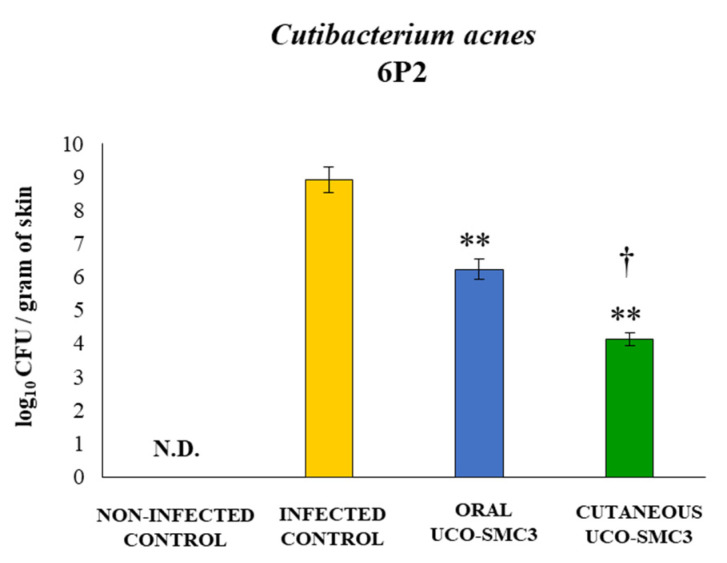
Effect of *Weissella viridescens* UCO-SMC3 on the resistance to *Cutibacterium acnes* skin infection. Mice were divided into three groups: with no UCO-SMC3 treatment (control group); treated topically (10^8^ bacterial cells/mL in sterile PBS on the back) with the UCO-SMC3 strain (cutaneous UCO-SMC3 group) or treated orally (10^8^ bacterial cells/mL in sterile PBS per gavage) with the UCO-SMC3 strain (oral UCO-SMC3 group). *W. viridiscens* UCO-SMC3 was administered daily for 10 days. On day 11, these mice and the untreated control group were challenged with *C. acnes* (subdermal injection of 10^8^ CFU). Pathogen cell counts in skin samples were determined 5 days after *C. acnes* infection. Statistically different compared to the infected control group (**) *p* < 0.01. Statistically different compared to the oral UCO-SMC3 group (†) *p* < 0.05. N.D.: not detected.

We next aimed to evaluate whether *W. viridescens* UCO-SMC3 could exert beneficial effects against *C. acnes* infection in vivo. For this purpose, a mice model of skin infection with the clinical isolate *C. acnes* 6P2 was developed. In this model, two different treatments with the UCO-SCM3 strain were assessed: oral (oral UCO-SMC3 group) and topical (cutaneous UCO-SMC3 group) administrations before the challenge with *C. acnes* (Supplementary Figure S1). As shown in Figure 5, *C. acnes* was not detected in skin samples of non-infected mice. In contrast, control infected mice had high *C. acnes* loads in skin samples suggesting that the pathogen successfully established steady intradermal colonization in the skin of BALB/c mice following injection. In addition, it was observed that both oral UCO-SMC3 and cutaneous UCO-SMC3 groups had significantly lower *C. acnes* counts in skin samples when compared to infected control mice. Of note, the topical administration of *W. viridescens* UCO-SMC3 was more efficient than the oral treatment to reduce the skin *C. acnes* loads (Figure 5).

### 3.6. Immunomodulatory Activity of W. viridescens UCO-SMC3

We aimed to assess whether the beneficial effect induced by the UCO-SCM3 strain in the in vivo model of *C. acnes* 6P2 infection was related to differential modulation of the immune response. First, we evaluated the number of blood leukocytes (Supplementary Figure S5) and serum cytokines (Supplementary Figure S6) in UCO-SCM3 treated mice before the challenge with *C. acnes* in order to evaluate the effect of the probiotic administration only. No significant differences were found between the oral UCO-SMC3 and cutaneous UCO-SMC3 groups and the control mice when the numbers of blood leucocytes, neutrophils, monocytes, or lymphocytes were compared (Supplementary Figure S5). The levels of serum TNF-α, IL-1β, IL-4, and IL-17 were similar in all the experimental groups (Figure Supplementary S6). However, mice treated with *W. viridescens* UCO-SMC3 by the oral route showed IFN-γ and IL-10 levels that were significantly higher than controls. In addition, mice in the cutaneous UCO-SMC3 group had concentrations of serum IFN-γ that were higher than controls.

**Figure 6:**
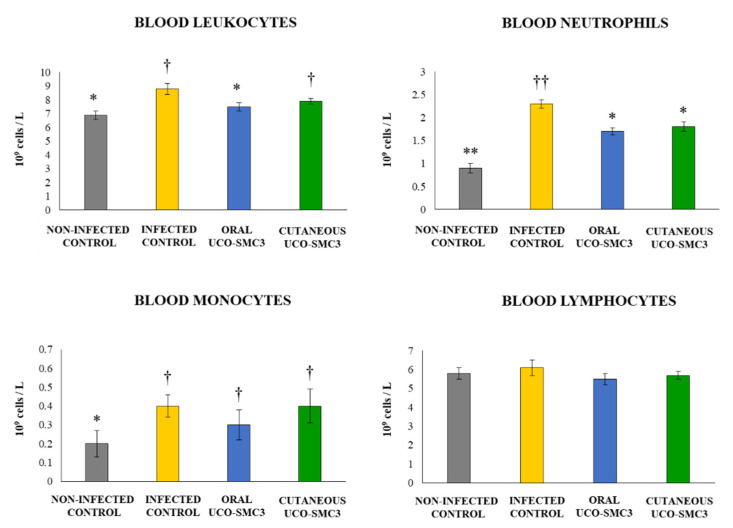
Effect of *Weissella viridescens* UCO-SMC3 on the immune response against *Cutibacterium acnes* skin infection. Mice were divided into three groups: with no UCO-SMC3 treatment (control group); treated topically (10^8^ bacterial cells/mL in sterile PBS on the back) with the UCO-SMC3 strain (cutaneous UCO-SMC3 group) or treated orally (10^8^ bacterial cells/mL in sterile PBS per gavage) with the UCO-SMC3 strain (oral UCO-SMC3 group). *W. viridiscens* UCO-SMC3 was administered daily for 10 days. On day 11, these mice and the untreated control group were challenged with *C. acnes* (subdermal injection of 10^8^ CFU). Leukocytes, neutrophils, monocytes, and lymphocytes counts in blood were determined 5 days after *C. acnes* infection. Statistically different compared to the infected control group (*) *p* < 0.05, (**) *p* < 0.01. Statistically different compared to the non-infected control group (†) *p* < 0.05, (††) *p* < 0.01.

As shown in Figure 6, the challenge of untreated control mice with the opportunistic pathogen significantly increased the blood leucocytes counts. This increment was produced by the increases of both blood neutrophils and monocytes, while the numbers of blood lymphocytes remained similar to those found in the non-infected control group. Mice in the cutaneous UCO-SMC3 group had values of blood leucocytes that were similar to those found in the control group. The increment was related to both blood neutrophils and monocytes although the number of neutrophils was significantly lower than the control group (Figure 6). On the other hand, mice in the oral UCO-SMC3 group had significantly lower levels of blood leucocytes when compared to controls. This difference was produced by blood neutrophils only since the number of blood monocytes was similar to control mice (Figure 6).

**Figure 7:**
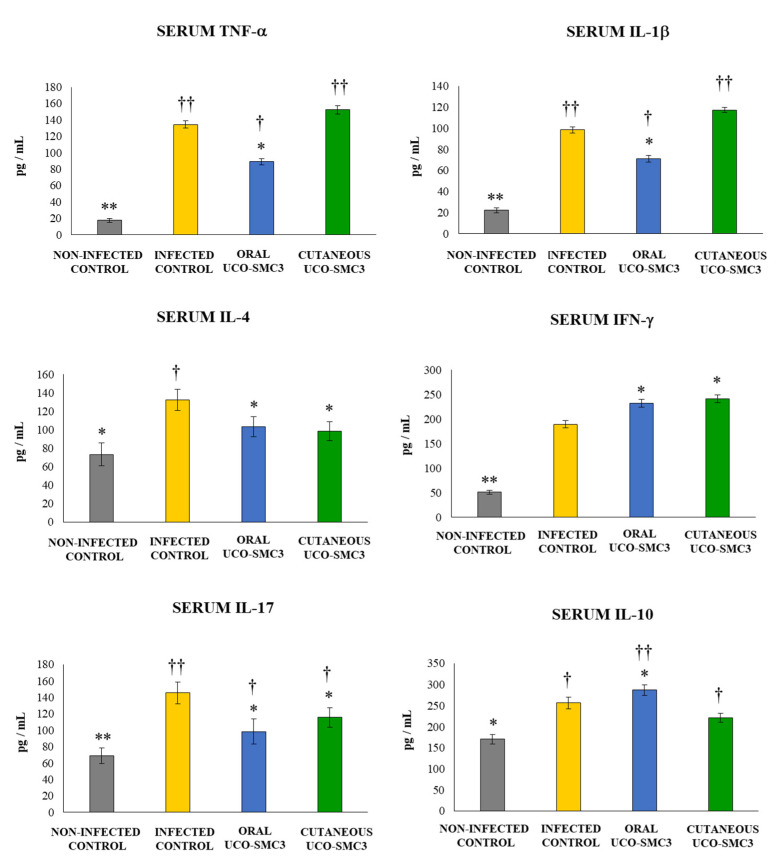
Effect of *Weissella viridescens* UCO-SMC3 on the immune response against *Cutibacterium acnes* skin infection. Mice were divided into three groups: with no UCO-SMC3 treatment (control group); treated topically (10^8^ bacterial cells/mL in sterile PBS on the back) with the UCO-SMC3 strain (cutaneous UCO-SMC3 group) or treated orally (10^8^ bacterial cells/mL in sterile PBS per gavage) with the UCO-SMC3 strain (oral UCO-SMC3 group). *W. viridiscens* UCO-SMC3 was administered daily for 10 days. On day 11, these mice and the untreated control group were challenged with *C. acnes* (subdermal injection of 10^8^ CFU). Tumor necrosis factor α (TNF-α), interferon-γ (IFN-γ), and interleukin 1β (IL-1β), IL-4, IL-10, and IL-17 concentrations in serum were determined 5 days after *C. acnes* infection. Statistically different compared to the infected control group (*) *p* < 0.05, (**) *p* < 0.01. Statistically different compared to the non-infected control group (†) *p* < 0.05, (††) *p* < 0.01.

We also evaluated the levels of several cytokines in the serum of infected mice (Figure 7). The challenge with *C. acnes* induced increases in the levels of serum TNF-α, IFN-γ, IL-1β, IL-4, and IL-17 in all the experimental groups. However, mice treated with *W. viridescens* UCO-SMC3 showed a different cytokine profile in terms of their serum concentration when compared to control animals. Mice in the oral UCO-SMC3 group had levels of the inflammatory cytokines TNF-α and IL-1β that were significantly lower than controls. In contrast, the levels of these two cytokines in the cutaneous UCO-SMC3 group were not different from controls (Figure 7). The serum IFN-γ concentrations in both the oral and the cutaneous UCO-SMC3 groups were significantly higher than controls. In addition, it was observed that mice treated with *W. viridescens* UCO-SMC3 by either the oral or the topical routes had levels of serum IL-4 and IL-17 that were significantly lower than control mice (Figure 7).

Variations in the levels of the immunoregulatory cytokine IL-10 were also analyzed. The infectious challenge with *C. acnes* enhanced the levels of serum IL-10 in all the experimental groups (Figure 7). Mice in the oral UCO-SMC3 group had levels of IL-10 that were significantly higher than controls. In contrast, the levels of this immunoregulatory cytokine in the cutaneous UCO-SMC3 group were not different from controls (Figure 7).

Finally, we aimed to study the effect of *C. acnes* infection and *W. viridescens* UCO-SMC3 treatments in the immune cell populations of facial (FLN) and axillary (ALN) lymph nodes, which drain the infection zone in our experimental model. For this purpose, the variations in CD11c^+^MHC-II^+^ antigen-presenting cells (APCs), CD3^+^CD4^+^ T cells, and CD4^+^CD25^+^ activated T cells were evaluated by flow cytometry in both lymphoid organs.

**Figure 8:**
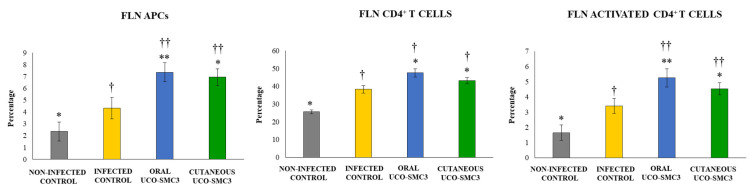
Effect of *Weissella viridescens* UCO-SMC3 on the immune response against *Cutibacterium acnes* skin infection. Mice were divided into three groups: with no UCO-SMC3 treatment (control group); treated topically (10^8^ bacterial cells/mL in sterile PBS on the back) with the UCO-SMC3 strain (cutaneous UCO-SMC3 group) or treated orally (10^8^ bacterial cells/mL in sterile PBS per gavage) with the UCO-SMC3 strain (oral UCO-SMC3 group). *W. viridiscens* UCO-SMC3 was administered daily for 10 days. On day 11, these mice and the untreated control group were challenged with *C. acnes* (subdermal injection of 10^8^ CFU). Variations in CD11c^+^MHC-II^+^ antigen-presenting cells (APCs), CD3^+^CD4^+^ T cells, and CD4^+^CD25^+^ activated T cells in facial lymph nodes (FLN) were determined 5 days after *C. acnes* infection. Statistically different compared to the infected control group (*) *p* < 0.05, (**) *p* < 0.01. Statistically different compared to the non-infected control group (†) *p* < 0.05, (††) *p* < 0.01.

**Figure 9:**
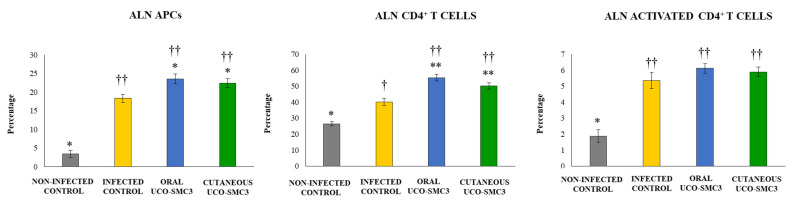
Effect of *Weissella viridescens* UCO-SMC3 on the immune response against *Cutibacterium acnes* skin infection. Mice were divided into three groups: with no UCO-SMC3 treatment (control group); treated topically (10^8^ bacterial cells/mL in sterile PBS on the back) with the UCO-SMC3 strain (cutaneous UCO-SMC3 group) or treated orally (10^8^ bacterial cells/mL in sterile PBS per gavage) with the UCO-SMC3 strain (oral UCO-SMC3 group). *W. viridiscens* UCO-SMC3 was administered daily for10 days. On day 11, these mice and the untreated control group were challenged with *C. acnes* (subdermal injection of 10^8^ CFU). Variations in CD11c^+^MHC-II^+^ antigen-presenting cells (APCs), CD3^+^CD4^+^ T cells, and CD4^+^CD25^+^ activated T cells in axillary lymph nodes (ALN) were determined 5 days after *C. acnes* infection. Statistically different compared to the infected control group (*) *p* < 0.05, (**) *p* < 0.01. Statistically different compared to the non-infected control group (†) *p* < 0.05, (††) *p* < 0.01.

The challenge with *C. acnes* increased the percentages of CD11c^+^MHC-II^+^ APCs in all FLN (Figure 8) and ALN (Figure 9) experimental groups, with changes in ALN more notorious than in FLN. The percentages of CD11c^+^MHC-II^+^ APCs in FLN and ALN in both the oral and the cutaneous UCO-SMC3 groups were significantly higher than controls.

*C. acnes* infection also induced increases in the percentages of CD3^+^CD4^+^ T cells, and CD4^+^CD25^+^ T cells in all the experimental groups, in both FLN (Figure 8) and ALN (Figure 9). The percentages of the two T cell populations in FLN and ALN in both the oral and the cutaneous UCO-SMC3 groups were significantly higher than controls.

### 3.7. Pilot Test

**Figure 10:**
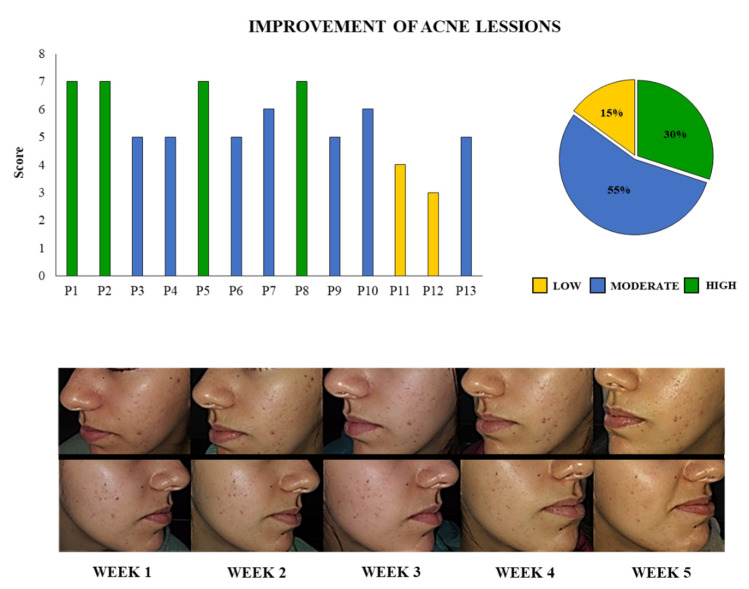
Evaluation of acne lesions evolution after the application of the cream containing the probiotic strain *Weissella viridescens* UCO-SMC3 in 13 volunteers. The score was determined considered grading to estimate the extent of involvement and lesion counting. The improvement of the lesions from the beginning to the end of the study was quantified considering a high (7 or 8 points), moderate (5 or 6 points), mild (3 or 4 points), or no (1 or 2 points) reduction of inflammation and extent of involvement, as described in details in materials and methods. The percentage of patients in each improvement group is shown. In addition, the photographic record of a patient with high improvement throughout the 5 weeks of study is shown.

The trial was conducted through 5 weeks of application of the probiotic UCO-SCM3 cream in 13 volunteers with acne and 5 volunteers without acne. In order to quantify the clinical examination of all the patients, a score was determined as described in the Materials and Methods. The score with a minimum of 2 (no reduction of inflammation and lesions counts) and a maximum of 8 points (high reduction of inflammation and lesions counts) was calculated for each patient (Figure 10). Seven patients (55%) had a moderate improvement of acne lesions (scores 5 and 6) while 4 patients (30%) presented a high improvement of skin lesions. Of note, only 2 patients (15%) had a low improvement of the lesions. Figure 10 also shows the photographic record of a volunteer with acne and the evolution during the 5 weeks of treatment. A noticeable change in the acne lesions and in the hydration of the skin can be observed during the course of the studied period because of the application of the prototype cream containing the UCO-SMC3 strain. A significant decrease in the inflammation of the lesions can be observed in weeks 4 and 5. Moreover, some of the acne lesions were completely healed. It was also observed that the UCO-SMC3 cream also helped to reduce spots and scars caused by acne.

It should be noted that in volunteers with healthy skin, no evident adverse effects such as inflammation or allergic reactions were observed after the application of the UCO-SMC3 cream for 5 weeks, indicating that the prototype UCO-SMC3 cream is safe for people with healthy skin.

## 4. Discussion

*C. acnes* has long been considered a commensal bacterium of the skin, however, its involvement in various infections has led to its emergence as an opportunistic pathogen. Of note, the proliferation of specific phylotypes of this bacterium and its ability to stimulate skin inflammation has been shown to be involved in acne vulgaris development [46]. The inflammatory response induced by *C. acnes* is mediated by the pattern recognition receptor Toll-like receptor-2 (TLR2) expressed in keratinocytes and APCs [47]. In vitro studies demonstrated that upon the *C. acnes* challenge, the mitogen-activated protein kinase (MAPK) and nuclear factor-kB (NF-kB) pathways are activated leading to the production of TNF-α, IL-8, IL-6, and IL-1β by HaCaT cells [48] and THP-1 macrophages [49]. In line with those findings, it was shown that the *C. acnes*-induced inflammatory response promotes the accumulation of neutrophils [50] and lymphocytes [51] in the sebaceous follicles, where they can promote the breakdown of the follicular wall, stimulating further inflammation. These two important aspects of acne, bacterial proliferation, and detrimental inflammation, have led to the search for treatments that can reduce the growth of *C. acnes* and diminish the generation and amplification of the inflammatory response [52]. Then, the development of safe therapeutic alternatives with strong antibacterial and immunomodulatory activities is highly desirable for the treatment of acne. One promising approach is the use of probiotic microorganisms, which efficiently inhibit the growth of pathogens and modulate host immune responses (reviewed in Lunjani et al. [1]). In this regard, we demonstrated here that *W. viridescens* UCO-SMC3, originally isolated from the slime of a garden snail, possesses the ability to antagonize *C. acnes* and modulate the immune system.

Recent studies reported differences in the gut microbiome of patients with acne suggesting a role for these microbial populations in the development of the disease [7,53]. Interestingly, it was demonstrated that acne patients harbored diminished diversity and increased *Bacteroidetes*:*Firmicutes* ratio in the gut [54]. It was suggested that the intestinal bacterial dysbiosis found in patients with acne would enhance intestinal permeability, leading to the release of microbial molecules into the circulation that promote inflammation contributing to acne development [55,56]. Thus, the use of oral probiotic interventions for the treatment of acne has gained significant attention over the past decade [1,57]. Clinical trials described improvement of acne by orally administered probiotics alone and in combination with standard treatments [58,59,60]. The administration of the mixture of the probiotic bacteria *Lactobacillus delbrueckii* LB-5, *Lacticaseibacillus acidophilus* NAS, and *Bifidobacterium bifidum* Malyot during 12 weeks was as effective as minocycline to reduce acne lesions [59]. Of note, fewer side effects were observed in the probiotic group than in patients receiving only minocycline. Similarly, a study revealed that patients receiving oral *Lacticaseibacillus rhamnosus* SP1 for 12 weeks exhibited significant improvement of their acne lesions compared with the placebo control group [58]. More recently, the double-blind, placebo-controlled randomized study performed by Kim et al. [56] demonstrated that the oral administration of *Lactiplantibacillus plantarum* CJLP55 to patients with acne during 12 weeks improved acne lesion count and grade, decreased sebum triglycerides, and increased skin hydration. Those beneficial effects were associated with an improvement in intestinal bacterial dysbiosis.

Taking into account these antecedents, one of the aims of this work was to investigate whether the oral administration of *W. viridescens* UCO-SMC3 improved the resistance to *C. acnes* infection and differentially modulated the immune response triggered by the bacterial challenge. For this purpose, we first performed in vitro studies in order to evaluate the ability of the UCO-SMC3 to resist gastrointestinal conditions since probiotic microorganisms should be able to overcome the low pH of the gastric juice and the detergent effect of the bile salts to reach the intestinal immune cells in a viable physiological state. Our functional experiments demonstrated that *W. viridescens* UCO-SMC3 has these characteristics. Furthermore, our preliminary studies in Caco-2 cells demonstrated that the UCO-SCM3 strain efficiently adheres to intestinal epithelial cells (unpublished results). It was shown that the intestinal porcine isolate *W. viridescens* MYU 208 is capable of efficiently adhering to porcine intestinal mucin. Of note, using a receptor overlay analysis to evaluate adhesins in the PBS extracts of the MYU 208 strain, it was found that three proteins had adhesin functions: GroEL, enolase, and the elongation factor Tu [61]. The genomic analysis of *W. viridescens* UCO-SMC3 indicates the presence of GroEL chaperonin (WP_124942787.1), enolase (WP_124943655.1), and the elongation factor Tu (WP_124943809.1) in its genome. In addition, the ssp5 gene for the agglutinin receptor that is able to bind sialic acid residues of the salivary agglutinin in a calcium-dependent manner was also found in the UCO-SMC3 genome [21], giving further evidence to support the ability of this strain to colonize, at least temporally, in the intestinal tract.

In order to demonstrate the potential beneficial effects of orally administered *W. viridescens* UCO-SMC3, we next used a mice model of acne for in vivo studies. Most of the studies using mice to evaluate *C. acnes* infection usually inject the pathogen into the animal’s ears [48,62,63]. *C. acnes* inoculated subcutaneously into the back of the ears induce ear swelling, redness, and erythema [48], which are associated with the local and systemic production of TNF-α, IL-6, and IL-8 [63] as well as the infiltration of CD45^+^Ly6G^+^ neutrophils and CD45^+^F4/80^+^ macrophages into the infected tissue [62]. Although this model is useful for studies of therapeutic options for acne, it has limitations associated with the small size of the animals’ ears. Because of the frailty of the mouse ear, the appearance of secondary changes due to inflammatory reaction is limited. In addition, it is not possible to measure easily the bacterial load after the challenge or to evaluate the role of the local microbiota. An alternative to this model is the injection of *C. acnes* into the dorsal skin to develop inflammatory nodules [36,37,64]. It was shown that the intensity of the acute inflammatory response induced after *C. acnes* infections depends on the mouse strain [38]. While in HR-1 mice, *C. acnes* infection generates epidermal hyperplasia and the formation of secondary microcomedones [64] and such reaction processes were less remarkable in BALB/c mice [65]. In fact, HR-1 mice develop acne-like inflammatory nodules on their backs following *C. acnes* challenge, which can be observed macroscopically. In contrast, BALB/c, do not develop such visible macroscopic changes [65]. Nevertheless, *C. acnes* is able to successfully establish steady intradermal colonization in the skin of BALB/c mice following injection inducing a prominent inflammatory response [37]. In line with those previous findings, we showed here that the injection of the clinical isolate *C. acnes* 6P2 in the back of BALB/c mice did not induce a remarkable development of nodules and, therefore, we were not capable of macroscopically quantifying the lesions. However, we were able to perform *C. acnes* counts in infected skin samples demonstrating that the pathogens persist after 5 days. Moreover, a remarkable induction of inflammatory response was detected as observed by the increases in blood leukocytes counts, serum inflammatory cytokines, and changes in immune cell population in FLN and ALN. Interestingly, mice treated with *W. viridescens* UCO-SMC3 before the challenge with *C. acnes* had significantly lower blood neutrophils, and serum TNF-α, IL-1β, and IL-17 levels. In addition, mice orally treated with the UCO-SMC3 strain had enhanced levels of serum IFN-γ as well as CD11c^+^MHC-II^+^, CD3^+^CD4^+^, and CD4^+^CD25^+^ cell numbers in FLN and ALN. This reduction in inflammation and improvement of the Th1 response was correlated with decreased levels of *C. acnes* in the skin samples in UCO-SMC3-treated mice. It was shown that the incubation of skin explants with *C. acnes* phylotype IA1 significantly upregulates the expression of IL-6, IL-8, and IL-17 when compared with the stimulation of phylotypes II or III [66]. In addition, it was observed that *C. acnes* phylotype IA1 stimulates strong Th1 and Th17 responses, while phylotypes associated with healthy skin induce low Th1 and Th17 responses and high production of IL-10 [3,67]. In line with these findings, it was shown that immunoregulatory cytokines are of key importance for normal cutaneous homeostasis. In human skin, immunoregulatory cytokines were described to modulate fibroblasts and APCs functions [68] while in mouse skin they were reported to establish immune tolerance to commensals [69] and to participate in wound healing [70]. In our hands, the oral administration of *W. viridescens* UCO-SMC3 significantly increased serum IL-10 indicating the ability of the bacterial treatment to promote anti-inflammatory mechanisms.

Our results show that orally administered *W. viridescens* UCO-SMC3 differentially modulates the inflammatory response, the Th1/Th17 balance, and the levels of IL-10 improving the resistance of mice to *C. acnes* infection. Unfortunately, we cannot provide here a mechanism through which the UCO-SMC3 strain, through its interaction with the gastrointestinal tract, beneficially modulates the systemic and skin immune responses triggered by *C. acnes* infection. It has been proposed that orally administered probiotics would exert their beneficial effects in distal sites from the intestine through four probable mechanisms, which are not mutually exclusive: (a) the mobilization of immune cells from the gut, (b) the release and absorption of microbial molecules that can impact immune receptors in non-intestinal tissues, (c) the release of cytokines and growth factors produced in the intestinal mucosa into the blood that acts systemically or in distal tissues, and (d) the metabolic reprograming of immune cells by microbial metabolites that are adsorbed in the intestine. The exact nature of the mechanism(s) used by orally administered *W. viridescens* UCO-SMC3 to modulate the skin’s immunity remains to be determined.

Commensal microorganisms inhabiting a certain ecological niche have the possibility to interact with pathogens inhibiting their colonization and growth [1,71]. Commensal bacteria can passively occupy the binding sites of a niche, thus impeding the colonization of pathogens [57]. Commensals may also consume nutrients reducing their bioavailability, secrete antimicrobial factors that synergize with antimicrobial peptides produced by the host, produce factors that interfere with the virulence signaling pathways of their competitors, and modulate the immune system [1,71]. These mechanisms are particularly relevant for commensal bacteria of the skin that protect against pathogens. In this regard, it was shown that *Staphylococcus epidermidis*, one of the most abundant commensal species in the skin, controls the proliferation of *C. acnes* by producing acids through the fermentation of glycerol synthetized naturally by the skin [23]. It was also demonstrated that *S. epidermidis* controls *S. aureus* growth in the skin directly by producing antimicrobials, and indirectly through the improvement of the tight junction barrier function and the modulation of the local immune system [72]. Those findings have stimulated the study of topically administered probiotics for controlling acne [73].

Our in vitro studies demonstrated the remarkable capacity of the UCO-SMC3 strain to inhibit the growth of *C. acnes* and also *S. aureus*, which is another microorganism associated with acne [74]. These findings are in line with some studies that proposed *W. viridescens* strains as probiotics, mainly due to their antimicrobial activity. Among more than one hundred LAB strains isolated from fermented food samples, *W. viridescens* WM33 showed a strong inhibitory effect against *Salmonella* spp. [75]. The cell-free culture supernatants of the WM33 strain inhibit *Salmonella* serovars but the precise molecules involved in this effect were not investigated. In addition, it was reported that *W. viridescens* C1 had a strong antimicrobial effect on *Listeria monocytogenes* and that this effect was induced by the production of acid, H_2_O_2_, and antimicrobial compounds of proteinaceous nature [76]. Similarly, our results indicate that the inhibitory effect of *W. viridescens* UCO-SMC3 on *C. acnes* and *S. aureus* would be mediated mainly by lactic acid and H_2_O_2_ since we were not able to demonstrate the presence of the bacteriocins pediocin, plantarazin A, sakasin P, acidocin, salivaricin B, or bacteriocin ABP118 by genetic and genomic studies. We also showed here that *W. viridescens* UCO-SMC3 has a remarkable ability to adhere to keratinocytes, which is in line with our previous in silico studies demonstrating the presence of two genes of collagen adhesins (*cna1* and *cna2*) and an extracellular matrix-binding protein gene (*ebh*) in the UCO-SMC3 genome [21]. Those factors would promote bacterial attachment to keratinocytes allowing the bacteria to compete for binding sites with *C. acnes* and *S. aureus*. Our results allow us to affirm with certainty that these two properties are also verified in vivo since topically administered *W. viridescens* UCO-SMC3 significantly reduced the *C. acnes* counts in skin lesions of mice. Of note, although the topical administration of the UCO-SMC3 was significantly more efficient than the oral route in reducing *C. acnes* counts in infected mice, lower immunological changes were observed with the topical administration than in orally treated animals. Reduction of blood neutrophils and serum IL-17, as well as enhancement of IFN-γ, CD11c^+^MHC-II^+^, and CD3^+^CD4^+^ cell numbers in FLN and ALN were found in topically treated mice as observed for the oral treatment. However, the oral treatment was more efficient to improve the numbers of activated CD4^+^CD25^+^ T cells and was the only treatment able to increase serum IL-10. The distinct microbial loads that the skin immune system must face in both experimental groups could explain these differences. In animals treated topically with *W. viridescens* UCO-SMC3, its direct inhibitory effect would reduce the number of pathogenic bacteria at the site of the infection, inducing less activation of the local immune response. Kinetic studies that evaluate the *C. acnes* loads and the immunological changes at different time points after the infectious challenge would be of great importance to clarify the differences between both UCO-SMC3 treatments.

In addition to the studies in the experimental animal model, our pilot clinical study provides additional evidence of the potential for *W. viridescens* UCO-SMC3 to be used as a probiotic for the treatment of acne. Although the number of trials assessing the effect of topical probiotic and prebiotic interventions in skin disease is limited [1,57], interesting studies have been published in relation to acne treatment. Twenty-nine acne female patients treated with an oil-in-water formulation containing *L. plantarum* twice daily for 2 months reduced erythema and acne lesion size [77]. In addition, the application of the glucomannan hydrolysates as prebiotics twice daily for about 6 weeks improved disease severity in 26 female patients with acne [78]. Similarly, the application of the cream containing the UCO-SMC3 strain in 13 volunteers with acne significantly enhanced the scores that quantified disease improvement. Moreover, the administration of the UCO-SCM3 cream to healthy volunteers did not induce any detrimental effect, which was in line with our in vitro and genomic studies that did not detect virulence factors in *W. viridescens* UCO-SMC3. Although this small study involving only young female individuals of the population derived from a single community prevents extrapolation of the current results to a broader scale, the results presented here encourage further mechanistic and clinical investigations to characterize *W. viridescens* UCO-SMC3 as a topical probiotic for acne treatment.

## 5. Conclusions

To the best of our knowledge, we were the first in describing the isolation of LAB from the slime of the garden snail *Helix aspersa* Müller and investigating those microorganisms as potential probiotics for the skin. One of the isolated strains, *W. viridescens* UCO-SMC3, demonstrated in silico some characteristics that allowed us to speculate that this bacterium could be used as a probiotic against skin infections [21]. In this work, our in vitro and in vivo results have confirmed our previous hypothesis by showing that the UCO-SMC3 strain can resist adverse gastrointestinal conditions, antagonize with *C. acnes*, and modulate the immune response against the skin pathogen. Both the oral and topical administration of the UCO-SCM3 strain was capable of reducing the replication of *C. acnes* and beneficially modulating the immune response. Of note, orally administered *W. viridescens* UCO-SMC3 induced more remarkable changes in the immune response to *C. acnes* than the topical treatment. However, the topical administration of *W. viridescens* UCO-SMC3 was more efficient than the oral treatment to reduce pathogen bacterial loads in the skin, and effects probably related to its ability to inhibit and antagonize the adhesion of *C. acnes*. Furthermore, a pilot study in acne-volunteers demonstrated the capacity of the UCO-SMC3 strain to reduce acne lesions when topically administered.

Early and effective treatment for acne is crucial not only for inhibiting the growth of pathogenic microbes but also for interrupting the microbial-triggered inflammatory response. Then, acne treatment requires both anti-bacterial and anti-inflammatory measures. Our results show that *W. viridescens* UCO-SMC3 could be an interesting candidate to achieve both effects. Clinical trials assessing the effect of the UCO-SMC3 strain administered orally, topically, or a combination of both with larger samples and greater power are necessary to conclusively demonstrate its beneficial effects as well as to characterize the safety, doses, and treatment durations that are most effective.

## Figures and Tables

**Table 1:** Antibiotic susceptibility profile of *Weissella viridescens* UCO-SMC3.

Antibiotic	Concentraion (μg)	*W. viridescens* UCO-SMC3
Streptomycin	10	S
Etrithromycin	15	S
Amikacin	30	MS
Gentamicin	10	S
Ampicilin	10	MS
Cefuroxim	30	S
Penicillin G	10	S
Sulfatrimethoprim	25	R
Cefotaxin	30	S
Amoxicillin	10	S
Levofloxacin	5	S
Chloramphenicol	30	S
Clarithromycin	15	S
Neomicin	30	S
Ciprofloxacin	5	R
Rifampicin	5	S
Vancomycin	30	MS
Tetracycline	30	S

Susceptibility is expressed as no inhibition (R); inhibition of 7–16 mm (MS); 16–25 (S) inhibition; inhibition > 25 mm (SS) (Georgieva, 2008).

## Data Availability

Data is contained within the article or supplementary material.

## Supplementary Materials

The following are available online at https://www.mdpi.com/article/10.3390/microorganisms9071486/s1, Figure S1: Experimental protocol for the evaluation of the effect of *Weissella viridescens* UCO-SMC3 in *Cutibacterium acnes* infection in mice, Figure S2: adhesion of *Weissella viridescens* UCO-SMC3 to HaCat epidermal keratinocytes, Figure S3: production of H_2_O_2_ by *Weissella viridescens* UCO-SMC3; Figure S4: Detection of bacteriocin ABP118 in *Weissella viridescens* UCO-SMC3 and *Ligilactobacillus salivarius* LGM14476, Figure S5: Effect of *Weissella viridescens* UCO-SMC3 on blood leucocytes counts. Mice were divided into three groups: with no UCO-SMC3 treatment (control group); treated topically (10^8^ bacterial cells/mL in sterile PBS on the back) with the UCO-SMC3 strain (cutaneous UCO-SMC3 group) or treated orally (10^8^ bacterial cells/mL in sterile PBS per gavage) with the UCO-SMC3 strain (oral UCO-SMC3 group). *W. viridiscens* UCO-SMC3 was ad-ministered daily during 10 days. On day 11, leukocytes, neutrophils, monocytes and lymphocytes counts in blood were determined. Figure S6: Effect of *Weissella viridescens* UCO-SMC3 on blood cytokines. Mice were divided into three groups: with no UCO-SMC3 treatment (control group); treated topically (10^8^ bacterial cells/mL in sterile PBS on the back) with the UCO-SMC3 strain (cutaneous UCO-SMC3 group) or treated orally (10^8^ bacterial cells/mL in sterile PBS per gavage) with the UCO-SMC3 strain (oral UCO-SMC3 group). *W. viridiscens* UCO-SMC3 was administered daily during 10 days. On day 11, TNF-α, IFN-γ, IL-1β, IL-4, IL-10 and IL-17 concentrations in serum were determined. Statistically different compared to the control group (*) *p* < 0.05.
