# Supplementary material for: Characterization of Weissella viridescens UCO-SMC3 as a Potential Probiotic for the Skin: Its Beneficial Role in the Pathogenesis of Acne Vulgaris
Source: Microorganisms. 2021 Jul 13;9(7):1486. doi: 10.3390/microorganisms9071486 (PMC8307422; doi:10.3390/microorganisms9071486)

Figure S1: Experimental protocol for the evaluation of the effect of *Weissella viridescens* UCO-SMC3 in *Cutibacterium acnes* infection in mice,

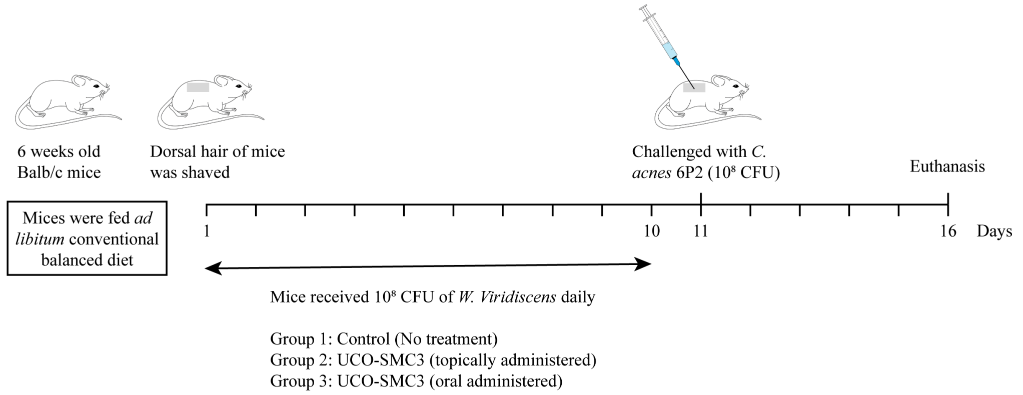

Figure S2: adhesion of *Weissella viridescens* UCO-SMC3 to HaCat epidermal keratinocytes,

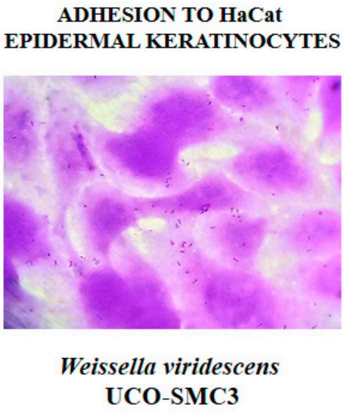

Figure S3: production of H_2O_2 by *Weissella viridescens* UCO-SMC3;

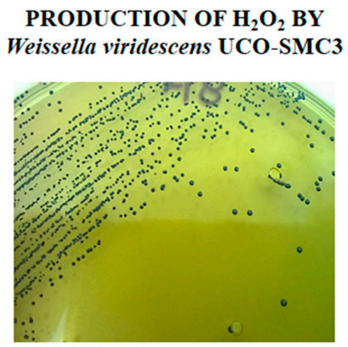

Figure S4: Detection of bacteriocin ABP118 in *Weissella viridescens* UCO-SMC3 and *Ligilactobacillus salivarius* LGM14476,

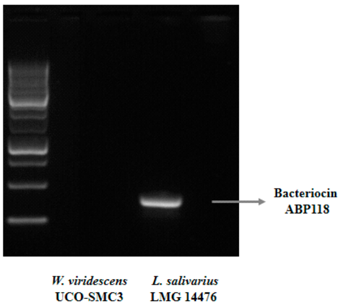

Figure S5: Effect of *Weissella viridescens* UCO-SMC3 on blood leucocytes counts. Mice were divided into three groups: with no UCO-SMC3 treatment (control group); treated topically (10^8 bacterial cells/mL in sterile PBS on the back) with the UCO-SMC3 strain (cutaneous UCO-SMC3 group) or treated orally (10^8 bacterial cells/mL in sterile PBS per gavage) with the UCO-SMC3 strain (oral UCO-SMC3 group). *W. viridiscens* UCO-SMC3 was administered daily during 10 days. On day 11, leukocytes, neutrophils, monocytes and lymphocytes counts in blood were determined.

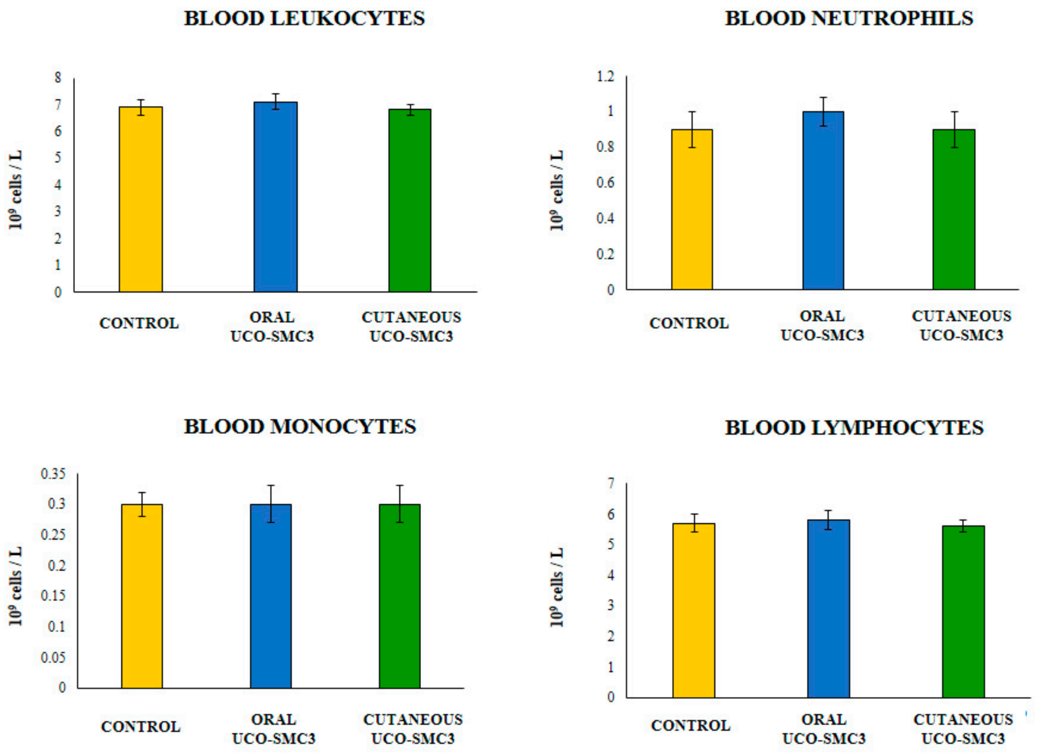

Figure S6: Effect of *Weissella viridescens* UCO-SMC3 on blood cytokines. Mice were divided into three groups: with no UCO-SMC3 treatment (control group); treated topically (10^8 bacterial cells/mL in sterile PBS on the back) with the UCO-SMC3 strain (cutaneous UCO-SMC3 group) or treated orally (10^8 bacterial cells/mL in sterile PBS per gavage) with the UCO-SMC3 strain (oral UCO-SMC3 group). *W. viridiscens* UCO-SMC3 was administered daily during 10 days. On day 11, TNF-α, IFN-γ, IL-1β, IL-4, IL-10 and IL-17 concentrations in serum were determined. Statistically different compared to the control group (*) $p < 0.05$.

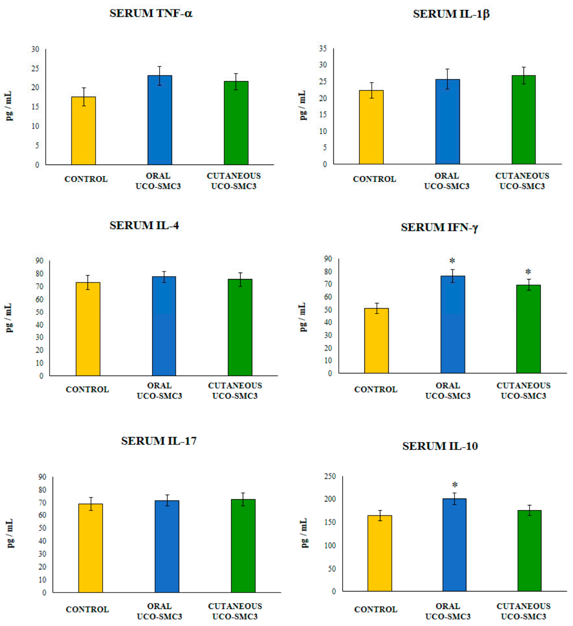

Supplement: Supplementary file 1 [file microorganisms-09-01486-s001.zip › microorganisms-1261150-supplementary.pdf]
